# A Case of Hypertrophic Pulmonary Osteoarthropathy in Both Upper and Lower Extremities: A Rare Involvement

**DOI:** 10.4274/mirt.78941

**Published:** 2018-06-07

**Authors:** Berna Okudan, Nazım Coşkun, Pelin Arıcan, Rıza Şefizade, Seniha Naldöken

**Affiliations:** 1 Ankara Numune Training and Research Hospital, Clinic of Nuclear Medicine, Ankara, Turkey

**Keywords:** Hypertrophic pulmonary osteoarthropathy, lung cancer, upper extremity

## Abstract

Hypertrophic pulmonary osteoarthropathy (HPOA) is a paraneoplastic manifestation of gastric and, more frequently, lung carcinomas. It is characterized by extremity pain, clubbing, arthritis and periostitis of the long bones. Periostitis is the hallmark of HPOA and can be revealed with bone scintigraphy. Whole-body bone scintigraphy (WBBS) is very sensitive during the active lesion period and WBBS findings usually precede that of plain radiography. WBBS can also show improvement in the first 6 months following treatment, thus making it an important technique in the management and follow-up of these patients. While HPOA findings are usually seen in the lower extremities, involvement of both upper and lower extremities is a rare condition. In this case report, it is aimed to present findings of a 67-year-old male patient with lung cancer and complaint of extremity pain. We report on this patient to draw attention to HPOA of both upper and lower extremities.

## Abstract (tr)

Hipertrofik pulmoner osteoartopati (HPOA), mide ve özellikle akciğer kanserlerinde görülebilen bir paraneoplastik sendromdur. Çomak parmak, artrit, ekstremite ağrısı ve uzun kemiklerde periostit ile karakterizedir. Periostit HPOA’nın ayırıcı bulgularındandır ve sintigrafi ile gösterilebilir. Sintigrafik bulgular çoğunlukla radyolojik bulgulardan önce ortaya çıkar. Tüm vücut kemik sintigrafisi (TVKS) aktif lezyon döneminde oldukça duyarlıdır. TVKS ile tedavi sonrası ilk 6 ayda bulgularda gerileme gösterilebilir. Dolayısıyla TVKS, bu hastaların tanı ve takibinde önemli bir yer tutar. HPOA bulguları çoğunlukla alt ekstremitede görülür; hem üst hem alt ekstremite tutulumu nadir görülen bir durumdur. Bu olgu takdimi, ekstremite ağrısı ile başvuran akciğer kanseri hastalarında, hem alt hem üst ekstremitede nadiren görülen HPOA tablosuna dikkat çekmek amacıyla sunulmaktadır.

## Figures and Tables

**Figure 1:**
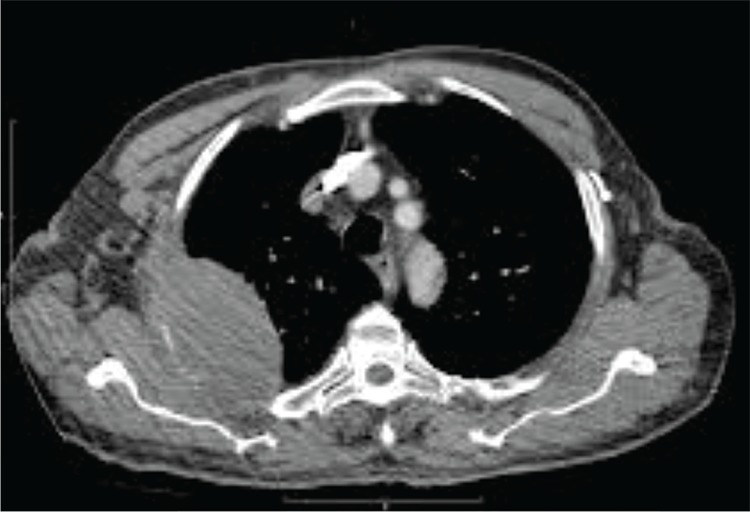
A 67-year-old male patient suffering from chest pain, cough, shortness of breath and extremity pain was referred to the department of radiology for imaging. Computed tomography of the chest showed a large mass in the right lung and biopsy cytology results were positive for adenocarcinoma. The patient was then referred to the department of nuclear medicine for whole-body bone scintigraphy (WBBS) due to extremity pain.

**Figure 2:**
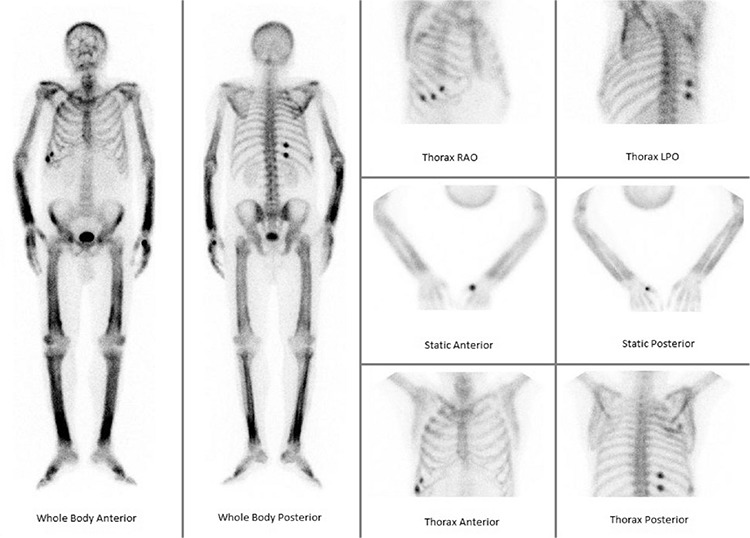
The WBBS showed non-homogeneous cortical uptake in bilateral upper and lower extremity bones, consistent with hypertrophic pulmonary osteoarthropathy (HPOA). Multiple focal spots were also seen on right hemithorax costae, possibly due to trauma. HPOA is a paraneoplastic manifestation of gastric and, more frequently, lung carcinomas. It is characterized by extremity pain, clubbing, arthritis and periostitis of the long bones. HPOA findings in lower extremities have been previously reported (1,2,3). However, involvement of both upper and lower extremities is a rare condition. Periostitis is the hallmark of HPOA and can be revealed with bone scintigraphy (4). WBBS is very sensitive during the active lesion period and WBBS findings usually appear before radiography findings. WBBS can also display the improvement within the first 6 months following treatment, thus making it an important technique in the management and follow-up of these patients.
